# Pulmonary histoplasmosis mimicking recurrent lung adenocarcinoma: A case report

**DOI:** 10.1016/j.rmcr.2026.102484

**Published:** 2026-08-14

**Authors:** Mauro Geller, Marcos Knobel, Marcelo Kalichsztein, Felipe Carbonieri, Regina C. Schechtman, David S. Lewi, Spyros G.E. Mezitis

**Affiliations:** a Immunology-Microbiology Department, Medical School UNIFESO, Rio de Janeiro, Brazil; b Clinical Immunology Department, Carlos Chagas Postgraduation Medical Institute, Rio de Janeiro, Brazil; c Cardiology – Internal Medicine, Hospital Israelita Albert Einstein, São Paulo, Brazil; d Pneumology Department, São Vicente Clinic, Rio de Janeiro, Brazil; e Thoracic Department, Felippe Mattoso Clinic, Rio de Janeiro, Brazil; f Dermatology Department, State University of Rio de Janeiro, Brazil; g Infectology Department, Hospital Israelita Albert Einstein, São Paulo, Brazil; h Weill Cornell Medicine-Presbyterian Hospital, New York, USA

**Keywords:** Histoplasmosis, Pulmonary nodule, Lung adenocarcinoma, FDG-PET, Granulomatous inflammation, Oncologic surveillance, Endemic mycosis, Case report

## Abstract

Pulmonary histoplasmosis, caused by the dimorphic fungus *Histoplasma capsulatum*, can present as solitary pulmonary nodules with granulomatous inflammation that radiologically and metabolically mimic malignant lesions, posing a significant diagnostic challenge in patients with a history of lung cancer. We report a case of a 69-year-old asymptomatic male with a prior left upper lobe adenocarcinoma resected in 2019, who presented with a new growing, hypermetabolic nodule in the left lower lobe during routine oncologic surveillance in December 2025. Sequential computed tomography (CT) demonstrated interval growth, and positron emission tomography (PET-CT) revealed intense fluorodeoxyglucose (FDG) uptake (SUVmax 7.2), strongly suggesting recurrence. Video-assisted thoracoscopic segmentectomy was performed; histopathological examination revealed chronic necrotizing granulomatous inflammation, with Grocott methenamine silver staining confirming small yeast forms consistent with *Histoplasma* spp. No malignancy was identified. This case underscores the diagnostic pitfall of fungal infections mimicking cancer recurrence in endemic regions and the indispensable role of histopathological confirmation prior to initiating systemic oncologic therapy.

## 1 Introduction

Histoplasmosis is an endemic mycosis caused by the thermally dimorphic fungus *Histoplasma capsulatum*, prevalent in the Americas, including Brazil, where soil contaminated with bird or bat droppings serves as the primary reservoir [1,2]. In immunocompetent individuals, infection is typically asymptomatic or self-limiting; however, chronic pulmonary forms can manifest as solitary nodules or cavitary masses with necrotizing granulomatous inflammation [2]. These lesions frequently exhibit computed tomography (CT) and positron emission tomography (PET) features that overlap significantly with malignancy, including spiculated or irregular borders, interval growth, and increased fluorodeoxyglucose (FDG) uptake [3]. This radiological and metabolic mimicry is particularly consequential in patients undergoing oncologic surveillance for prior lung cancer, where any new or enlarging nodule raises concern for recurrence or a second primary malignancy [4]. We present a case in which pulmonary histoplasmosis was strongly suspected to represent recurrent lung adenocarcinoma based on clinical and imaging criteria, ultimately confirming the indispensable role of histopathological evaluation in this clinical scenario.

## 2 Case presentation

A 69-year-old male with a history of left upper lobe lung adenocarcinoma (diagnosed and resected via anatomical trisegmentectomy in March 2019) presented for routine annual surveillance imaging in December 2025. The patient was entirely asymptomatic, reporting no cough, dyspnea, hemoptysis, weight loss, fever, or other systemic symptoms. He had no significant comorbidities, was a lifelong non-smoker, and resided in Brazil, a geographic region endemic for histoplasmosis. Oncologic follow-up imaging was performed per standard surveillance protocol for resected stage I lung adenocarcinoma.

## 3 Radiological findings

**Fig. 1:**
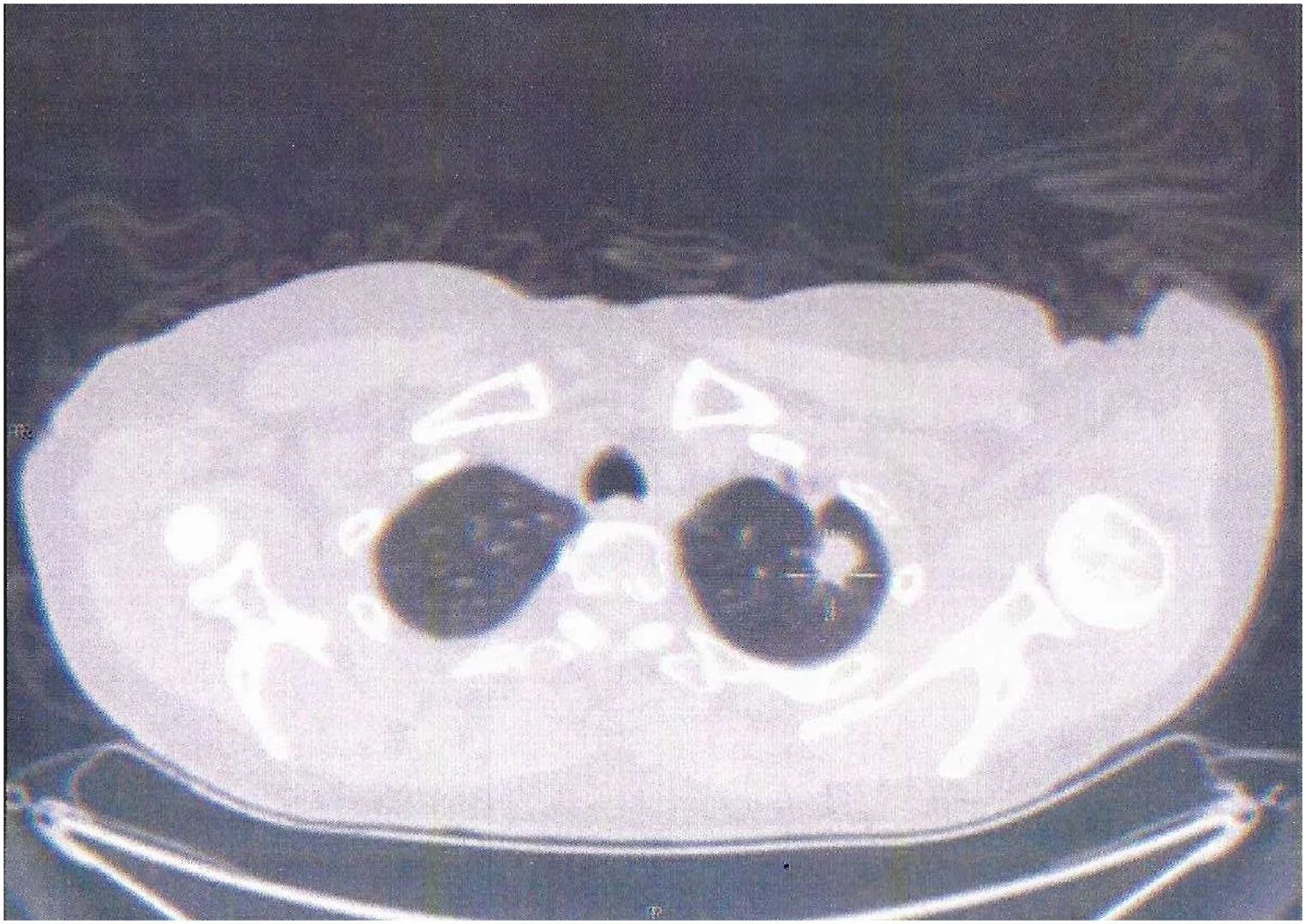
PET-CT with 18F-FDG (December 20, 2025).

**Fig. 2:**
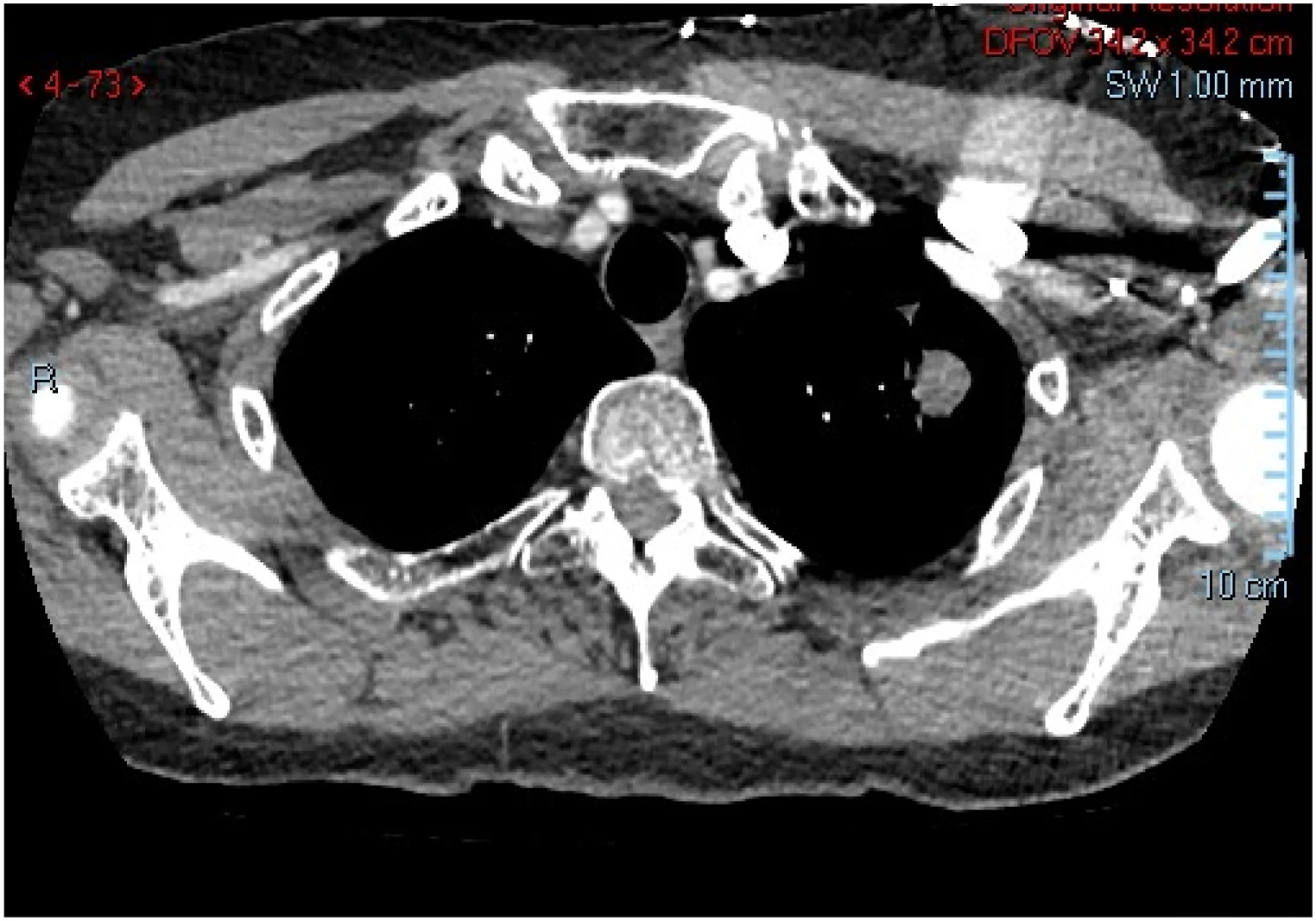
Angio-CT Thorax with 3D reconstructions for surgical planning (January 7, 2026).

• **CT Thorax (December 18, 2025):** Revealed an increase in size and change in morphology of a nodule in the superior segment of the left lower lobe (from 1.5 × 0.6 cm to 2.4 × 1.8 cm compared to January 21, 2025). The nodule appeared solid, elongated, irregular, and mildly heterogeneous, with surrounding reticular opacities extending to the pleural surface. Additional findings included regression of a small ground-glass opacity in the same lobe, new mild ground-glass opacities, centrilobular nodules suggestive of small airway filling, and bronchial impaction, predominantly in basal segments of the left lower lobe—favoring inflammatory changes. Small pleural effusion on the left and no lymphadenopathy were noted. Impression: Suspicious evolutionary behavior warranting further investigation.
• **PET-CT with 18F-FDG (December 20, 2025) –** Fig. 1: Demonstrated heterogeneous hypermetabolism in the left lower lobe nodule (SUVmax 7.2, delayed SUVmax 7.7), with focal peripheral medial uptake and heterogeneous contrast enhancement. The nodule was stable in size (2.4 × 1.8 cm). A small irregular opacity in the right upper lobe showed mild uptake (SUVmax 1.1, delayed 1.4), indeterminate. Persistent inflammatory-appearing opacities in both lungs lacked significant metabolic activity. Secondary finding: Focal prostatic hypermetabolism (SUVmax 6.7), indeterminate. Additional CT findings: Post-surgical changes in left upper lobe, small left pleural effusion, rare indeterminate micronodules, renal cysts, colonic diverticula, vascular calcifications, and right proximal humerus osteosynthesis.

• **Brain MRI (December 29, 2025):** Discrete hyperintense foci on T2/FLAIR in periventricular white matter and right frontal/parietal lobes, non-enhancing, suggestive of gliosis from microangiopathy. No evidence of cerebral metastasis.
• **Angio-CT Thorax with 3D Reconstructions for Surgical Planning (January 7, 2026) –** Fig. 2: Confirmed the solid nodule measuring 2.0 cm (volume 3.7 mL) in segment S6 of the left lower lobe, 4.2 cm from the S6/S9 intersegmental plane. Bronchial anatomy: B6 single and topical; common basal trunk originating B8, B9, B10. Arterial: A6 single and topical; common basal trunk originating A8+9 and A10. Venous: V6, V8+9, V10 draining into left inferior pulmonary vein. Segmental volumetry: S6 (613 mL, 31%), S8 (157 mL, 8%), S9 (328 mL, 16%), S10 (477 mL, 24%). Other findings: No pleural effusions, post-surgical changes, atelectatic bands, small non-calcified nodules (up to 0.6 cm, some new), tiny air cyst in right lower lobe, no mediastinal lymphadenopathy, normal vascular calibers, mild spinal degeneration, and right humeral fixation.

Postoperative chest X-rays (January 10–22, 2026) showed evolving changes: Initial subcutaneous emphysema, pigtail drain placement, reduced left lung volume, apical pneumothorax, and atelectatic opacities resolving over time; mild left pleural effusion increasing slightly then stabilizing; bilateral vascular prominence; normal cardiothoracic index.

## 4 Laboratory and functional evaluations

Preoperative assessments were largely unremarkable:

**Table:** 

Test	Key Results	Reference Range	Interpretation
Hemogram (December 29, 2025)	Erythrocytes 4.78 × 10^6/μL; Hemoglobin 15.2 g/dL; Hematocrit 44.0%; Leukocytes 6010/μL (neutrophils 67.8%, lymphocytes 21.6%, monocytes 8.2%, eosinophils 1.8%, basophils 0.6%); Platelets 280,000/μL	Normal ranges as per lab	Normal; no anemia, infection, or thrombocytopenia
Inflammatory Markers	ESR (VHS) 13 mm/1st hour	Inferior to 20 mm	Normal
Tumor Markers/Coagulation	PT 14.6 s (activity 83%); INR 1.11; aPTT 34.9 s (ratio 1.01); Fibrinogen normal	Normal ranges	No coagulopathy
Infectious Disease Tests	Aspergillus galactomannan index 0.04	<0.50 (negative)	Negative; no evidence of aspergillosis
Other	HbA1c 5.6%; Estimated average glucose 114 mg/dL; MCV 92.0 fL; MPV 10.2 fL	Normal	No diabetes or microcytosis

• **Aspergillus Galactomannan (December 29, 2025):** Negative (index 0.04). Notes emphasized serial testing in high-risk patients and potential false-positives from antibiotics, but irrelevant here.
• **Pulmonary Function Test (Spirometry):** No ventilatory disturbances; FEV1 and FVC normal; FEV1/FVC preserved.
• **Echocardiogram (December 29, 2025):** Normal cavity dimensions and wall thicknesses; preserved left ventricular systolic function (global and segmental); normal diastolic parameters; mild mitral and aortic valve thickening with mild regurgitation; normal inferior vena cava and pericardium.
• **Electrocardiogram (January 9, 2026):** Normal sinus rhythm; no abnormalities noted.

## 5 Surgical and pathological findings

The patient underwent video-assisted thoracoscopic segmentectomy of the superior segment (S6) of the left lower lobe on January 9, 2026, with lymph node sampling (stations 9, 10, 11).

• **Intraoperative Frozen Section:** Margins free; partial nodular evaluation showed intense chronic inflammation with necrosis, abscess areas, and granulomatous outlines. Awaited paraffin studies.
• **Final Pathology (January 15, 2026):**
  ➢ Segment A (left lower lobe): Chronic granulomatous inflammatory process with necrosis; architectural distortion from multiple granulomas with palisading/epithelioid histiocytes around necrotic centers; lymphoid aggregates; multinucleated giant cells (some Langhans-type); prior hemorrhage with hemosiderin; dystrophic calcifications; peribronchovascular chronic infiltrate; subpleural/interlobar fibrosis. Ziehl-Neelsen negative for acid-fast bacilli; Grocott positive for small yeast forms suggestive of **Histoplasma** spp. No malignancy.
  ➢ Lymph nodes: B (station 9) and C (station 10): Reactive lymphoid hyperplasia, no malignancy. D (station 11): Chronic granulomatous lymphadenitis with distorted architecture, multiple granulomas, epithelioid histiocytes, giant cells, central necrosis; Ziehl-Neelsen negative; Grocott positive for **Histoplasma** like yeasts. No malignancy.
  ➢ Macroscopy: 14 g segment (6.5 × 3.5 × 2.7 cm) with irregular white-yellowish firm nodule (2.0 cm), abutting pleura, 1.5 cm from resection margin.
  ➢ Immunohistochemistry: TTF-1 and Napsin-A positive in pneumocytes; pancytokeratin in epithelial cells; p40 in myoepithelial cells—consistent with granulomatous process, no tumor.

Postoperatively, the patient recovered well, with serial X-rays showing resolution of pneumothorax and effusion by late January 2026. No antifungal therapy details were provided, but correlation with clinical, microbiological, and serological data was recommended.

## 6 Discussion

This case illustrates a well-recognized but clinically consequential diagnostic pitfall: pulmonary histoplasmosis mimicking lung cancer recurrence in a patient with a prior resected adenocarcinoma [1,2]. The index nodule exhibited all classical imaging and metabolic features of malignancy interval growth of more than 50% in under 12 months, irregular morphology with reticular extensions to the pleural surface, and intense FDG avidity on PET-CT (SUVmax 7.2–7.7) [3]. Such findings, particularly in a patient with established oncologic history, appropriately raise immediate concern for recurrence or a second primary tumor and typically justify surgical evaluation [4].

The high FDG uptake observed in this case reflects the metabolic activity of activated macrophages, epithelioid histiocytes, and multinucleated giant cells that constitute the granulomatous response to *Histoplasma capsulatum*. Multiple series have demonstrated that granulomatous infections, including histoplasmosis, sarcoidosis, and tuberculosis, can produce SUVmax values exceeding 4.0—and in some cases exceeding 10.0 rendering PET-CT alone insufficient to exclude infectious etiology [3,5]. The absence of systemic inflammatory markers (normal ESR, preserved leukocyte count) and the negative Aspergillus galactomannan did not meaningfully reduce the probability of fungal infection, as histoplasmosis in immunocompetent hosts frequently elicits a contained, localized granulomatous response without detectable systemic inflammation or serological markers [1,6]. Urine *Histoplasma* antigen testing and serology (complement fixation and immunodiffusion) were not performed preoperatively [6,7], representing a potential area for improvement in the workup of indeterminate nodules in endemic regions particularly when tissue sampling carries procedural risk or the patient declines invasive staging.

From a management standpoint, the decision to proceed with surgical resection rather than CT-guided biopsy or bronchoscopic sampling was driven by the nodule's peripheral location in segment S6, its metabolic profile, the patient's acceptable pulmonary reserve, and his prior oncologic history. In clinical practice, guidelines support prompt tissue diagnosis for indeterminate nodules with high-risk features in cancer survivors; however, the choice between percutaneous biopsy and resection should be individualized [4]. In this case, the triple-phase imaging workup CT, PET-CT, and angio-CT with 3D reconstructions provided essential anatomical detail for the segmentectomy and confirmed surgical feasibility. Pathological examination remained the cornerstone of diagnosis: Grocott methenamine silver staining revealed small intracellular yeast forms consistent with *Histoplasma* spp., while Ziehl-Neelsen staining excluded mycobacterial infection and immunohistochemistry definitively ruled out malignancy. The involvement of lymph node station 11 with granulomatous lymphadenitis further supports a reactive infectious process. The documented need for antifungal therapy in this setting is debated: current guidelines from the Infectious Diseases Society of America (IDSA) suggest that chronic pulmonary histoplasmosis in immunocompetent patients who undergo complete surgical resection may not require antifungal consolidation [1], though clinical judgment and post-surgical microbiological correlation are essential. This case also highlights the broader epidemiological importance of raising awareness of fungal infections as oncological mimics in South American endemic zones, where clinical suspicion may be lower at academic centers less familiar with histoplasmosis.

## 7 Conclusion

Pulmonary histoplasmosis can closely simulate recurrent lung adenocarcinoma on both morphological and metabolic imaging, potentially leading to unnecessary or premature surgical intervention. In patients residing in or with recent travel history to endemic regions, fungal infection must remain in the differential diagnosis for indeterminate pulmonary nodules, even in the setting of prior malignancy. Preoperative workup should include serological and urinary antigen testing for *Histoplasma capsulatum* where feasible. Histopathological examination with fungal-specific stains (Grocott) and immunohistochemistry to exclude malignancy remains indispensable for definitive diagnosis. This case reinforces the critical value of a multidisciplinary approach integrating clinical epidemiology, advanced imaging, and pathological analysis to ensure accurate diagnosis and avoid the harms of misclassification in the oncologic surveillance setting.

## CRediT authorship contribution statement

**Mauro Geller:** Conceptualization, Data curation, Formal analysis, Project administration, Supervision, Writing – original draft, Writing – review & editing. **Marcos Knobel:** Conceptualization, Investigation, Methodology. **Marcelo Kalichsztein:** Data curation, Formal analysis, Investigation. **Felipe Carbonieri:** Conceptualization, Formal analysis, Investigation. **Regina C. Schechtman:** Investigation. **David S. Lewi:** Formal analysis, Investigation. **Spyros G.E. Mezitis:** Conceptualization, Data curation, Formal analysis.

## Patient consent

Written informed consent was obtained from the patient for publication of this case report and any accompanying images. Patient identifying information has been anonymized in accordance with institutional data protection policies.

## Ethics declaration

This study was approved by the Institutional Ethics Committee under CAAE n°95928626.1.0000.0251. All procedures performed in this study were in accordance with the ethical standards of the institutional and national research committee and with the 1964 Helsinki Declaration and its later amendments [8].

## Artificial intelligence disclosure

The authors used xAI's Grok 2025 and AIClaude (Anthropic, version claude-sonnet-4-6, 2025) to assist with manuscript editing, structural revision, scientific language refinement, and discussion expansion during the preparation of this manuscript. The AI tool did not contribute to the clinical care of the patient, the acquisition or interpretation of data, or the formulation of scientific conclusions. All content was critically reviewed, verified, and approved by the authors, who take full and sole responsibility for the accuracy, integrity, and scientific content of the final manuscript. This disclosure is made in accordance with the guidelines of the International Committee of Medical Journal Editors (ICMJE) and the Committee on Publication Ethics (COPE) regarding the use of artificial intelligence tools in scholarly publication.

## Declaration of competing interests

The authors declare no conflicts of interest relevant to this manuscript. No role in design/analysis/publication and no other conflicts.
